# Supplementary material for: Identification and classification of urban employment centers based on big data: A case study of Beijing
Source: PLoS One. 2024 May 24;19(5):e0299726. doi: 10.1371/journal.pone.0299726 (PMC11125475; doi:10.1371/journal.pone.0299726)
Supplement: Attachment — Submitted filename: Response to Reviewer 2.docx [file pone.0299726.s001.docx]

Response to Reviewer 2 Comments

**Point 1:** In the Methodology part, four methods were used in the manuscript: Shannon-Wiener Diversity Index, XGBoost, DBSCAN, K-means. Why are those methods chosen? How to justify their appropriateness? It is suggested to add some explanations or comparisons with other similar methods to make your method more convincing.

**Response 1:** Thank you very much for your comments. Your constructive comments are helpful to the logic of our article, and I have explained the corresponding methodology to study the innovative use of the Shannon Wiener Diversity Index, which describes biodiversity, to analyze employment center industry diversity. The Shannon Wiener Diversity Index is primarily used to measure species richness within a system and The Shannon-Wiener Diversity Index is used to measure the intra-system species richness and the homogeneity of the distribution of individuals across classes. DBSCAN is a density-based clustering algorithm in which the number of objects (points or other spatial objects) contained in a certain region of the clustering space is not less than a given threshold, and the algorithm can discover arbitrarily shaped clusters from a collection of data with noise, XGBoost is an integrated learning algorithm that builds a strong classifier by training multiple weak classifiers for supervised learning classification of users' places of residence and employment, etc. K-means is a common clustering algorithm that divides data points into K different clusters, each of which has similar characteristics, and since K means is an unsupervised learning algorithm that does not require a priori knowledge and can automatically classify employment centers based on their industrial diversity and the average commuting distance of employed people.

**Point 2:** It is recommended to draw a large flowchart to illustrate the role of these methods in different stages of the study. This will make it easier for readers to understand the authors’ intentions.

**Response 2:** Thank you very much for your comments. I have drawn flowcharts that show the data and methods used more clearly, and modified the language accordingly for good reading.

**Point 3:** The classification of employment centers is a little confusing, it could be that I missed some important detail, I would therefore suggest the authors elaborate on the significance of the classification, or analyze the functions or influencing factors of employment centers at different levels.

**Response 3:** Thank you very much for your comments, some background and summary discussions have been added to the article. For employment centers, the significance of classification is to better provide targeted planning for different types of employment centers. This paper mainly classifies employment centers in terms of their industries and the average commuting time of employment center's job seekers. On the one hand, the more homogeneous the industry of an employment center is, the less stability it is subject to, and of course, a single employment center will also produce aggregation effects (but it needs to have a dominant industry). On the other hand, the average commuting time of job seekers in employment centers affects their commuting accessibility, and from the perspective of urban planning, convenient transportation facilities need to be provided for job seekers. Therefore, different types of employment centers require different types of support and services. By classifying employment centers, these supports and services can be better located and provided.

**Point 4:** The index of commuting data, the authors described it in line 258 as follows: average commuting aggregation force, but actually the authors calculated the commuting time (Table 4) and in line 396, this index became average commuting distance. There are confusion and inconsistency for this index. Please clarify its definition and make it consistent throughout the text.

**Response 4:** Thank you very much for your comments. average commuting aggregation force is my translation error, this paper is based on the average commuting time of job seekers in job centers combined with the industrial diversity of job centers to classify job centers, the average commuting time of job seekers in job centers affects their commuting accessibility, from the perspective of urban planning, it is necessary to provide job seekers with From an urban planning perspective, it is necessary to provide convenient transportation facilities for job seekers. Thank you very much for pointing out the mistake I made, and I have revised the average commute time error in this paper.

**Point 5:** In terms of the organization of the paper, firstly, it is better to have a paragraph outlining the rest of the article at the end of the introduction Section. Besides, in the conclusion part, presenting the contributions and innovations of the article in a detailed and organized manner through several points is more recommended.

**Response 5:** Thank you very much for your suggestions and comments on the organization of the paper. The suggestions you mentioned are very practical and important to help the writer better organize and present the essay. Adding a paragraph outlining the rest of the article at the end of the introduction section can give the reader a better understanding of the structure and content of the article, and in the conclusion section, it is also very important to show the contributions and innovations of the article in a detailed and organized manner. This can help the reader to better understand the value and significance of the article.

Minor comments are as follows:

**Point 1:** Language of the manuscript-at-hand needs to be revisited, i.e., particularly, in terms of the sentence structure and the punctuation.

**Response 1:** Thank you very much for your comment. I have modified the language accordingly for a good read.

**Point 2:** Line 415, “The second type of employment centers” should be “The third type” to be logical.

**Response 2:** Thank you very much for your comment. It was my mistake and I have changed it to “The third type”.

**Point 3:** Maps in figure 1, 2, 4, 5, 6 need to be supplemented with scale bar.

**Response 3:** Thank you very much for your comment. I have added the scale bar in figure 1, 2, 4, 5, 6.

**Point 4:** Please supplement Figure 7 with some key to the symbols to illustrate the three types of employment centers you divide.

**Response 4:** Thank you very much for your comment. I have made modifications to Figure 7 and added a legend to illustrate the three types of employment centers.
